# Biological Monitoring of Metal Ions Released from Hip Prostheses

**DOI:** 10.3390/ijerph17093223

**Published:** 2020-05-06

**Authors:** Annamaria Nicolli, Andrea Trevisan, Isabella Bortoletti, Assunta Pozzuoli, Pietro Ruggieri, Andrea Martinelli, Alberto Gambalunga, Mariella Carrieri

**Affiliations:** 1 Department of Cardiac Thoracic Vascular Sciences and Public Health, University of Padova, 35128 Padova, Italy; andrea.trevisan@unipd.it (A.T.); isabella.bortoletti@aopd.veneto.it (I.B.); andrea.martinelli@unipd.it (A.M.); alberto.gambalunga@unipd.it (A.G.); mariella.carrieri@unipd.it (M.C.); 2 Department of Surgery, Oncology and Gastroenterology, University of Padova, 35128 Padova, Italy; assunta.pozzuoli@unipd.it (A.P.); pietro.ruggieri@unipd.it (P.R.)

**Keywords:** hip prostheses, metal ions, metal debris, biological monitoring

## Abstract

The aim of this study was to evaluate the levels of As, Be, Bi, Cd, Co, Cr, Cu, Hg, Mn, Ni, Pb, Se, Tl, V, and Zn, by inductively coupled plasma-mass spectrometry (ICP-MS) in the urine of two groups of patients with two different types of metal-on-metal (MoM) total hip prostheses (ASR DePuy^®^, group A, 25 patients; total Met-Met System Lima^®^, group B, 28 patients). The determination of metals reflected a steady-state release (group A: 9 years after surgery and group B: 6 years after surgery). The results obtained confirmed the increase of Co and Cr urinary levels in both group when compared with the reference values for the general population adopted by the Italian Society of Reference Values (SIVR). In particular, Co and Cr levels exceeded the threshold values in urine, respectively, of 30 μg and 21 µg, adjusted to creatinine based on the threshold in whole blood of 7 μg/L proposed by the Medicines and Healthcare Products Regulatory Agency (MHRA). Regarding the other investigated metals, significantly higher values were found in Group A than in Group B. These differences could be due to the type of hip prosthesis implanted, the longer period of time since the implantation, as well as many other factors such as diet, age, drug consumption, physical activity, or presence of dental fillings. The continuous monitoring over the years of metal concentrations in patients carrying a prosthesis could be useful to better identify the sources of these metals.

## 1. Introduction

Hip prosthesis implants are widely used in orthopedic surgery. Although most metal-on-metal (MoM) hip replacements are successful, some patients could suffer from serious secondary adverse effects due to their malfunctioning.

Metallosis is a post-surgical complication that results from the surface deterioration of MoM bearings in orthopedic implants accompanied by an inflammatory response that can involve both macrophage-induced cytotoxicity, stimulated by metal debris, and a type IV delayed hypersensitivity reaction to metal particles. There are numerous factors that can affect hip prosthesis malfunction and consequent erosion with release of metal particles, such as the type of implant, different construction materials, surgery procedure, angle of inclination of the acetabular components, increased physical activity. The release of metallic particles, mainly cobalt (Co) and chromium (Cr) as components of metal alloys, into the surrounding tissues causes the phenomenon called adverse reaction to metal debris (ARMD). In addition, very high concentrations of Co and Cr in biological fluids due to malfunction of hip prostheses in some patients are associated with serious systemic symptoms including neurological symptoms (blindness, deafness), cardiomyopathy, and hypothyroidism [1,2]. In fact, the removal of the malfunctioning MoM prosthesis leads to the decline of the circulating metals with improvement of these symptoms [3,4].

**Figure 1:**
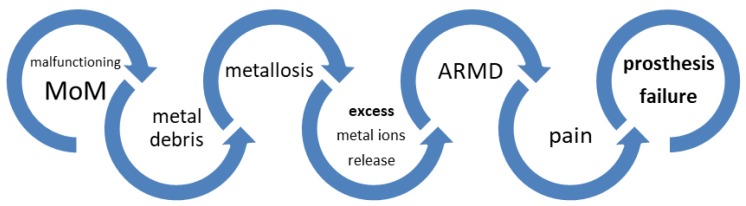
Events leading to loss of function of a metal-on-metal (MoM) hip replacement. ARMD, adverse reaction to metal debris.

Macroscopically, metallosis appears as a gray discoloration of the hip joint, which can subsequently be the origin of pain and instability in patients and of dysfunction of MoM hip arthroplasties (Figure 1). The greatest wear usually occurs during the first 1–2 years after surgery [5], which is followed by a low, but steady, wear over the subsequent years. The Medicines and Healthcare Products Regulatory Agency (MHRA) in Great Britain states that a concentration of metal ions in whole blood above 7 μg/L indicates a potential local tissue damage, and a progressive increase in metal ion levels must be considered as an early proof of implant loosening [6]. The replacement of malfunctioning MoM hip prostheses causes a quick and dramatic decrease of metal ion concentrations in whole blood and urine.

Numerous studies measured Co and Cr levels, the major constituents of the alloys used for hip prostheses, in patients with hip replacement [7,8,9], but only few studies focused on other metals that could be present in the alloys, even if in trace amounts [10,11]. 

The aim of this work was to investigate, by Inductively Coupled Plasma-Quadrupole Mass Spectrometry (ICP-QMS), the potential presence and release of metals other than Co and Cr in two groups of patients carrying two different MoM (Co-Cr) hip prostheses manufactured by different companies, who had not yet undergone revision.

## 2. Material and Methods

### 2.1. Patients

Two groups of patients were studied: the first group (Group A), implanted with ASR DePuy^®^ MoM total hip prostheses, was composed of 25 subjects (age at surgery 56.9 years, range 30–74 years). They were 19 males and 6 females. The second group (Group B), implanted with Lima^®^ MoM total Met-Met System hip prostheses, was composed of 28 patients (age at surgery 64.7, range 19–82). They were 11 males and 17 females. Table 1 summarized the characteristics of the studied subjects.

### 2.2. Ethics Statement

The research was based on data gathered according to recall program of Device Alert; therefore, evaluation by an ethics committee was not required. Informed consent was obtained for the anonymous treatment of the results for Group A and approved by the local Ethics Committee (#2561P) for Group B.

### 2.3. Urine Sampling and Analysis

Spot urine samples (the second urination in the morning) were collected into plastic sterile containers, immediately transferred into BD Vacutainer^®^ (REF 364918) by the needle located in the lid of the plastic container, and frozen at −20 °C until the time of analysis that was performed by an ICP-QMS (NexION 350X ICP-MS, Perkin Elmer, Waltham, MA, USA) system equipped with a collision system using helium as collision gas. To evaluate the transfer of material from the containers, tests were made with nitric acid 1% *v/v* (HNO_3_ Suprapur, Merck Millipore, Milan, Italy) kept in contact with the containers for 30 min and overnight. Analytical tests on the liquid gave a negative result (<LoD) for all the elements studied.

Seronorm Trace Elements Urine L-2 (SERO AS, Billingstad, Norway) and CPAchem QC 31 components (CPAchem Ltd., Stara Zagora, Bulgaria) were included as Internal Quality Controls (IQC) in each batch, and only data with ICQ values within the reference ranges were considered for the analysis. 

Our laboratory was also enrolled in the External Quality Assessment Schemes (EQAS) for the Occupational and Environmental Laboratory Medicine program conducted by the Italian Government at the time of this study, obtaining consistently satisfactory results.

For the analyses, 500 µL of each sample or IQC was dispensed into polypropylene assay tubes (Sarstedt Ag&Co, Numbrecht, Germany) with 4.5 mL of assay diluent composed of 1% *v/v* HNO_3_ in ultrapure water (obtained from a Milli-Q device IQ 7010, Merck Millipore, Milan, Italy) and 10 µL of IS-Mix as internal standard (1% *v/v* HNO_3_ solution containing 1 µg/L of Y, Re, Rh and 2 µg/L of Ge). 

Calibration standards were prepared by dilution from a custom stock solution (CPAchem Ltd., Stara Zagora, Bulgaria). The standard concentrations were in the range of 0.25–100 µg/L for Bi and Zn, in the range of 0.5–50 µg/L for As, and in the range 0.5–10 µg/L for all other metals. The samples with concentrations over the calibration curve were further diluted. The calibration standards, IQCs, and diluted samples were sequentially sampled using an S10 autosampler and introduced into the ICP-MS equipped with a glass type C nebulizer and a Glass cyclonic spray chamber. The NexION setup solution (1 µg/L Be, Ce, Fe, In, Li, Mg, Pb, U in 1% HNO_3_, Perkin Elmer, Waltham, MA, USA) was used for instrument setting. The counts per seconds (cps) data for the elements were normalized to the internal standards cps, and a calibration curve was plotted using ordinary linear regression in SyngiStix program (PerkinElmer, Waltham, MA, USA). The regression equation was then applied to the normalized elements cps for each sample and IQC to give the related concentrations. 

For quantification, the following analyte isotopes were used: ^75^As, ^9^Be, ^209^Bi, ^111^Cd, ^59^Co, ^52^Cr, ^63^Cu, ^202^Hg, ^55^Mn, ^60^Ni, ^208^Pb, ^78^Se, ^205^Tl, ^51^V, ^66^Zn. The method was validated in terms of precision, accuracy, and limit of detection. The inter-day precision of the method, expressed as CV% determined from the analysis of 3 independent samples at concentrations of 10 and 100 µg/L of added metals tested over 3 days, was lower than 16.4% for all metals. The accuracy, calculated using the Seronorm Trace Elements Urine L-2, ranged from 87% to 106%.

The estimated Limit of Detection (LOD) for each element, calculated as mean plus 3 times the standard deviation of the concentration of 10 blank samples, was: As, 0.044 µg/L; Be, 0.006 µg/L; Bi, 0.003 µg/L; Cd, 0.038 µg/L; Co, 0.026 µg/L; Cr, 0.053 µg/L; Cu 0.160 µg/L; Hg 0.030 µg/L; Mn 0.045 µg/L; Ni, 0.026 µg/L; Pb, 0.353 µg/L; Se, 0.949 µg/L; Tl, 0.030 µg/L; V, 0.019 µg/L; Zn, 2.72 µg/L.

## 3. Results

Since various ionic species present in low concentrations in the alloys used for hip prostheses could be released and have adverse effects in patients, we investigated whether such metals were effectively released in our patients due to wear of the MoM hip replacement after a long period of implantation.

Table 2 summarizes the results for arsenic (As), beryllium (Be), bismuth (Bi), cadmium (Cd), copper (Cu), lead (Pb), manganese (Mn), mercury (Hg), nickel (Ni), selenium (Se), thallium Tl), vanadium (V), and zinc (Zn), in addition to Cr and Co, released in urine by the implants at medium- to long-term follow up, i.e., after 9 years from surgery for Group A and after 6 years from surgery for Group B. Table 2 reports also the reference values (5°–95° percentile) for the general adult population adopted by the Italian Society of Reference Values (SIVR) evaluated in subjects not occupationally exposed to metals, who are not active smokers and do not bear surgical prostheses [12].

As expected, Co and Cr concentrations were significantly higher with respect to the value range indicated by the SIVR. The levels of the other analyzed metals were, for both groups, within the SIVR ranges (Cu, Hg, Mn, Ni, and Tl) or slightly higher (Be, Bi, Cd, Pb, Se, V, and Zn), with the exception of As, whose concentration as higher. 

The comparison between the two groups showed, with the exclusion of Bi, Cd, and As, higher levels of the analyzed metals in the urine of Group A than in that of Group B, and this difference achieved statistical significance (Mann-Whitney test) for Be (*p* = 0.0096), Co (*p* = 0.001), Cr (*p* = 0.0039), Cu (*p* = 0.0069), Hg (*p* = 0.0006), Mn (*p* < 0.0001), Ni (*p* = 0.0002), Pb (*p* = 0.0009), Se (*p* = 0.0103), V (*p* = 0.0223), and Zn (*p* = 0.0083).

Moreover, both Co and Cr exceeded, in about 30% of cases in both groups, the previously proposed thresholds (Table 3) in urine of 30 μg for Co and 21 μg for Cr adjusted to creatinine (expressed in g), based on the threshold in whole blood of 7 μg/L established by the MHRA as the concentration associated with potential local tissue damage [13]. 

## 4. Discussion

Hip replacement surgery is the most successful and prevalent modality treatment for degenerative hip diseases. An expanding elderly population and recent improvements in orthopedics techniques have led to an increasing number of people with hip prosthesis around the world. Although new designs and innovative materials have been investigated in order to obtain more efficient and durable devices, increased concentrations of circulating metals have been reported in hip prosthesis bearers [14,15,16,17], causing a mounting concern regarding adverse local and systemic effects of elevated metal levels released form hip prosthesis implants. 

MoM hip prosthesis wear causes an increase of circulating metal ions [18], and the measurement of Co and Cr in whole blood (CoB, CrB) is commonly considered the best approach to monitor hip-replaced patients [19]. Co and Cr increase in whole blood is suggestive of MoM hip prostheses failure, with high specificity (90% and 92%, respectively, 89% together) but low sensitivity (49% and 38%, respectively, 32% together) when related to the threshold (7 μg/L) stated by the MHRA. Values of CoB higher than 19 μg/L and of CrB higher than 17 μg/L are more likely to be associated with metallosis [20]. On the other hand, CoB levels up to 300 μg/L have not been associated with adverse responses in humans [21]. 

In a previous study, we have shown that patients with MoM hip implants had an increased release of Co and Cr into the blood [13]. In particular, 93.1% of patients had CoB and CrB concentrations outside the range of reference values adopted by the SIVR. In addition, 47.2% and 29.2% of patients, respectively, showed CoB and CrB concentrations higher than the MHRA suggested limit of 7 μg/L. We also showed, based on the high correlation found between metal concentrations in whole blood and urine, that these measures in urine and blood are suitable to monitor MoM hip-replaced patients. Indeed, our data indicated that the measurement of metals in urine is a reliable and precise way to monitor metal ion release, especially when adjusted to creatinine, as previously suggested [22,23,24]. Accordingly, a threshold, equal to 30 µg/g of creatinine for Co and 21 µg/g of creatinine for Cr, has been proposed. The analyses of previous studies were carried out by atomic absorption spectrometry (AAS). However, AAS is not a multielement technique, and very low metal concentrations (parts per trillion, ppt) cannot be measured. ICP–MS could, instead, be considered one of the most powerful techniques for the determination of metals in biological fluids due to its multielement analysis capability, its low detection limits, and the small sample volume required. Since we found that the measurement of metals in urine is a reliable and precise way to monitor metal ions release [13], we decided to evaluate the potential steady-state release in urine of different metals in two patients populations implanted with different types of MoM total hip prostheses. All subjects enrolled in the study had a hip prosthesis implant surgery that had not undergone revision yet.

The urine concentrations of As, Be, Bi, Cd, Cu, Hg, Mn, Ni, Pb, Se, Tl, V, and Zn, in addition to Co and Cr, were investigated. Although the presence of all these metals in the Cr-Co alloy used for the two prostheses was not confirmed, we decided to analyze a large number of metals because the exact composition of the Cr-Co alloy was not known.

As expected, the results showed a significant increase of Co and Cr ions in the urine of both groups when compared with the values adopted by the SIVR, and in around 30% of patients, the concentrations of Co and Cr ions exceeded the proposed thresholds, suggesting a potential local tissue damage. No or slight modifications were observed for Be, Bi, Cd, Cu, Hg, Mn, Ni, Pb, Se, V, Tl, and Zn, suggesting that (a) the alloys used for the examined prostheses did not contain such metals or (b) their release time could be different with respect to those of Co and Cr. The concentration of As was higher than the SIVR value in both Group A and Group B (60% and 11%, respectively), but these values could be affected by various parameters such as diet. The SIVR values (expressed in µg/L) represent the urinary levels of metals in non-occupationally exposed Italian adult subjects. The exclusion criteria adopted to establish them were occupational exposure to metals, smoking, presence of surgical prosthesis and of chronic diseases requiring the use of drugs. 

A factor that limits the comparison of our values with the SIVR reference values is the different age of our studied subjects with respect to the age of the individuals included in the SIVR analysis. The SIVR reference values refer to 18–60-year-old subjects (mean 39.7 years), while the subjects in our study were 25–89 years old (age at metal ion measurement), with a mean of 60.9 years. 

Finally, it is important to note that, with the exclusion of Bi, Cd, and the aforementioned As, the patients in Group A, implanted with the ASR DePuy^®^ prosthesis, showed statistically significantly higher values for most of the investigated metals. These findings could be due to the type of hip prosthesis implanted or the period of time since the implantation (9 vs. 6 years) as well as to many other factors such as diet, age, drug consumption, physical activity, presence of dental fillings, or environmental exposure. A further follow up could be useful to confirm if the differences in urinary metal concentrations between the two groups are due to the prostheses.

## 5. Conclusions

Due to the metal transport function of biological fluids from prostheses surfaces to local and remote tissues and organs, the concentration of these elements in urine and blood could be used as a potential indicator of metal release from the implants. In this work, the concentration of arsenic, beryllium, bismuth, cadmium, chromium, cobalt, copper, lead, manganese, mercury, nickel, selenium, thallium, vanadium, and zinc were simultaneously determined in the urine of two groups of people with hip prosthesis by ICP-MS. With regard to Co and Cr, a significant increase of their urinary concentrations was confirmed in both groups, while, apart from As, the other metals tested showed concentrations within the SIVR range values or slightly higher. 

On the basis of the results, the elevated urinary Co and Cr concentrations and potentially also those of other metals as a result of metal release from MoM implants remain a concern because they may have adverse long-term biological effects, including local adverse effects, hypersensitivity, mutagenicity, teratogenicity, and carcinogenicity [25]. Thus, further studies, also in blood samples, as well as the continuous monitoring over the years of patients with hip prosthesis, are needed.

## Figures and Tables

**Table 1:** Characteristics of the groups.

Variable. as Mean (Range)	Group A			Group B		
	Total (*n* = 25)	Males (*n* = 19)	Females (*n* = 6)	Total (*n* = 28)	Males (*n* = 11)	Females (*n* = 17)
Age at surgery (years)	56.9 (30–74)	57.3 (38–68)	55.8 (30–74)	64.7 (19–82)	64.0 (19–79)	65.2 (33–82)
Age at metal ion measurement (years)	66.4 (44–84)	66.6 (48–76)	65.8 (44–84)	71.6 (25–89)	70.3 (25–86)	72.3 (40–89)
Interval between surgery and metal ion measurement (years)	8.9 (6–13)	8.7 (6–11)	9.3 (6–13)	6.4 (2–11)	6.3 (4–10)	6.5 (2–11)

**Table 2:** Concentration of metal ions in the urine of two groups of patients and reference values (µg/L) established by the Italian Society of Reference Values (SIVR).

Metal	Group A			Group B			SIVR Values
	Mean ± sd	Median	5°–95°	Mean ± sd	Median	5°–95°	5°–95°
**As**	37.0 ± 42.6	22.3	4.62–113.1	42.1 ± 80.9	11.9	1.4–242.3	nd–16.1
**Be**	0.023 ± 0.012	0.022	0.011–0.041	0.018 ± 0.013	0.012	0.006–0.042	<0.01–0.034
**Bi**	0.02 ± 0.02	0.01	0.002–0.034	0.02 ± 0.02	0.01	0.002–0.051	nd–0.03
**Cd**	0.44 ± 0.29	0.39	0.12–1.05	0.56 ± 0.55	0.40	0.13–1.53	0.1–0.9
**Co**	55.7 ± 41.5	60.8	5.01–108.5	20.6 ± 23.5	11.5	1.57–67.00	0.077–2.2
**Cr**	17.7 ± 11.8	15.8	5.4–42.8	9.9 ± 8.9	7.4	0.8–23.0	0.05–0.6
**Cu**	13.3 ± 5.2	10.4	8.7–22.5	9.6 ± 6.8	7.5	2.5–22.0	5.01–24
**Hg**	1.10 ± 0.93	0.92	0.13–2.88	0.48 ± 0.66	0.26	0.03–1.49	0.1–5
**Mn**	0.808 ± 0.316	0.792	0.39–1.41	0.273 ± 0.244	0.209	0.04–0.60	0.04–1.5
**Ni**	2.67 ± 0.89	2.84	1.24–3.80	1.41 ± 1.01	1.31	0.32–3.33	0.372–4.44
**Pb**	2.07 ± 0.90	2.15	0.98–3.74	1.20 ± 1.47	0.54	0.05–2.74	0.17–2.64
**Se**	35.4 ± 14.6	35.5	14.2–62.7	26.7 ± 22.7	18.9	6.5–78.6	nd–61.6
**Tl**	0.279 ± 0.310	0.215	0.12–0.42	0.186 ± 0.142	0.161	0.03–0.40	0.06–0.759
**V**	0.643 ± 0.507	0.527	0.30–1.13	0.443 ± 0.290	0.361	0.18–1.10	0.025–0.855
**Zn**	953 ± 438	825	408–1720	632 ± 621	343	44–1836	nd–1048

sd: standard deviation; nd: not detected.

**Table 3:** Urine Co and Cr mean values adjusted to creatinine.

	% Co Levels > 30 µg/g Creatinine	% Cr Levels > 21 µg/g Creatinine	% Co > 30 µg/g Creatinine and Cr Levels > 21 µg/g Creatinine (both)
Group A	72	32	28
Group B	39	36	32
